# Secretoglobin 3A2 eliminates human cancer cells through pyroptosis

**DOI:** 10.1038/s41420-020-00385-w

**Published:** 2021-01-15

**Authors:** Shigetoshi Yokoyama, Shun Nakayama, Lei Xu, Aprile L. Pilon, Shioko Kimura

**Affiliations:** 1 Laboratory of Metabolism, National Cancer Institute, National Institutes of Health, Bethesda, MD 20892 USA; 2 APCBio Innovations, Inc., Rockville, MD 20850 USA

**Keywords:** Non-small-cell lung cancer, Apoptosis

## Abstract

Non-canonical inflammasome activation that recognizes intracellular lipopolysaccharide (LPS) causes pyroptosis, the inflammatory death of innate immune cells. The role of pyroptosis in innate immune cells is to rapidly eliminate pathogen-infected cells and limit the replication niche in the host body. Whether this rapid cell elimination process of pyroptosis plays a role in elimination of cancer cells is largely unknown. Our earlier study demonstrated that a multi-functional secreted protein, secretoglobin (SCGB) 3A2, chaperones LPS to cytosol, and activates caspase-11 and the non-canonical inflammasome pathway, leading to pyroptosis. Here we show that SCGB3A2 exhibits marked anti-cancer activity against 5 out of 11 of human non-small cell lung cancer cell lines in mouse xenographs, while no effect was observed in 6 of 6 small cell lung cancer cell lines examined. All SCGB3A2-LPS-sensitive cells express syndecan 1 (SDC1), a SCGB3A2 cell surface receptor, and caspase-4 (CASP4), a critical component of the non-canonical inflammasome pathway. Two epithelial-derived colon cancer cell lines expressing SDC1 and CASP4 were also susceptible to SCGB3A2-LPS treatment. TCGA analysis revealed that lung adenocarcinoma patients with higher *SCGB3A2* mRNA levels exhibited better survival. These data suggest that SCGB3A2 uses the machinery of pyroptosis for the elimination of human cancer cells via the non-canonical inflammasome pathway, and that SCGB3A2 may serve as a novel therapeutic to treat cancer, perhaps in combination with immuno and/or targeted therapies.

## Introduction

Inflammasomes are multimeric protein complexes that function as one of the pattern recognition receptors (PPRs) to activate proinflammatory caspases^1^. While TLR4 (Toll-like receptor 4) is a well-known PPR-recognizing extracellular lipopolysaccharides (LPS), the inflammasome is a PPR which recognizes microbe-derived molecules present in cytosol and activates the pyroptosis pathway. The inflammatory caspases, CASP1 and CASP11 (the human orthologs CASP4/CASP5^2^), cause pyroptosis of innate immune cells, which show characteristic features of plasma membrane pore formation and the ensuing cell swelling^3–6^. CASP1 is activated by the canonical inflammasome pathway which then activates multiple substrates including the pro-inflammatory cytokines interleukin 1B (IL1B) and IL18^7^, while CASP11 is activated by the “non-canonical pathway”, which is elicited by direct binding of the intracellular LPS released from Gram-negative bacteria, independent of TLR4 signaling^2,8^.

The physiological significance of pyroptosis is that severely microbe-infected cells can be rapidly eliminated from the host, resulting in a limited milieu where infectious agents can thrive and expand their colonization^9^. Pyroptosis promotes pathogens clearance by acting as an alarm signal through release of IL1B/IL18, and other factors that attract immune cells to the infectious sites in the host body^3^.

Recent evidence demonstrates that the NLR family pyrin domain containing 3 (NLRP3) inflammasome has pro-tumorigenic effects for some cancer types, while for others it prevents tumor development, depending on a cancer context^10^. In human hepatocellular carcinoma cells (HCC), NLRP3 inflammasome components are either completely lost or downregulated^11^, while reconstitution of NLRP3 inflammasome components in HCC significantly inhibit the malignant features^12^. Further, *Casp11*^−/−^ mice are highly susceptible to the azoxymethane-dextran sodium sulfate model of colitis-associated cancer, suggesting the anti-tumor role of CASP11 and the non-canonical inflammasome pathway in colitis-associated cancer^13^. However, the anti-cancer roles of CASP11 (CSAP4/5 in human) and non-canonical inflammasome are largely unexplored and have remained controversial.

We recently reported that multi-functional small secreted protein, secretoglobin family 3A member 2 (SCGB3A2), also known as UGRP1 (uteroglobin-related protein 1)^14–16^ is a potent LPS-binding protein and works as a chaperone to deliver LPS into mouse Lewis lung carcinoma (LLC) cells^17^. LLC cells express abundant cell surface receptor syndecan-1 (SDC1), the primary heparan sulfate (HS) proteoglycan to which SCGB3A2 binds, resulting in internalization of the SCGB3A2 + LPS complex. The internalized LPS activates CASP11 through the non-canonical inflammasome pathway, leading to pyroptosis of carcinoma cells^17^. As a result, SCGB3A2 significantly inhibits the growth and metastasis of LLC cells in vivo in a mouse intravenous xenograft model. However, whether SCGB3A2 also inhibits human cancer cells growth by means of pyroptosis has not been explored yet.

In order to investigate whether SCGB3A2 inhibits metastatic growth of human cancer cells, a lung cancer intravenous metastasis model using NSG (non-obese diabetic (NOD) scid gamma) mice was employed. We found that SCGB3A2 exhibits a potent anti-tumorigenic effect in some cancer types, especially non-small-cell lung cancers (NSCLCs) and epithelial-derived colorectal cancers. These cells are derived from epithelial tissues, constantly exposed to various pathogens. Our results highlight a critical role for SCGB3A2 in eliminating cancer cells through the same mechanism used to eliminate pathogens. These results provide the possibility of using SCGB3A2 as a novel cancer treatment.

## Results

### Differential SDC1 and CASP4 expression patterns determine the effect of SCGB3A2 in human cancer cell lines

**Fig. 1:**
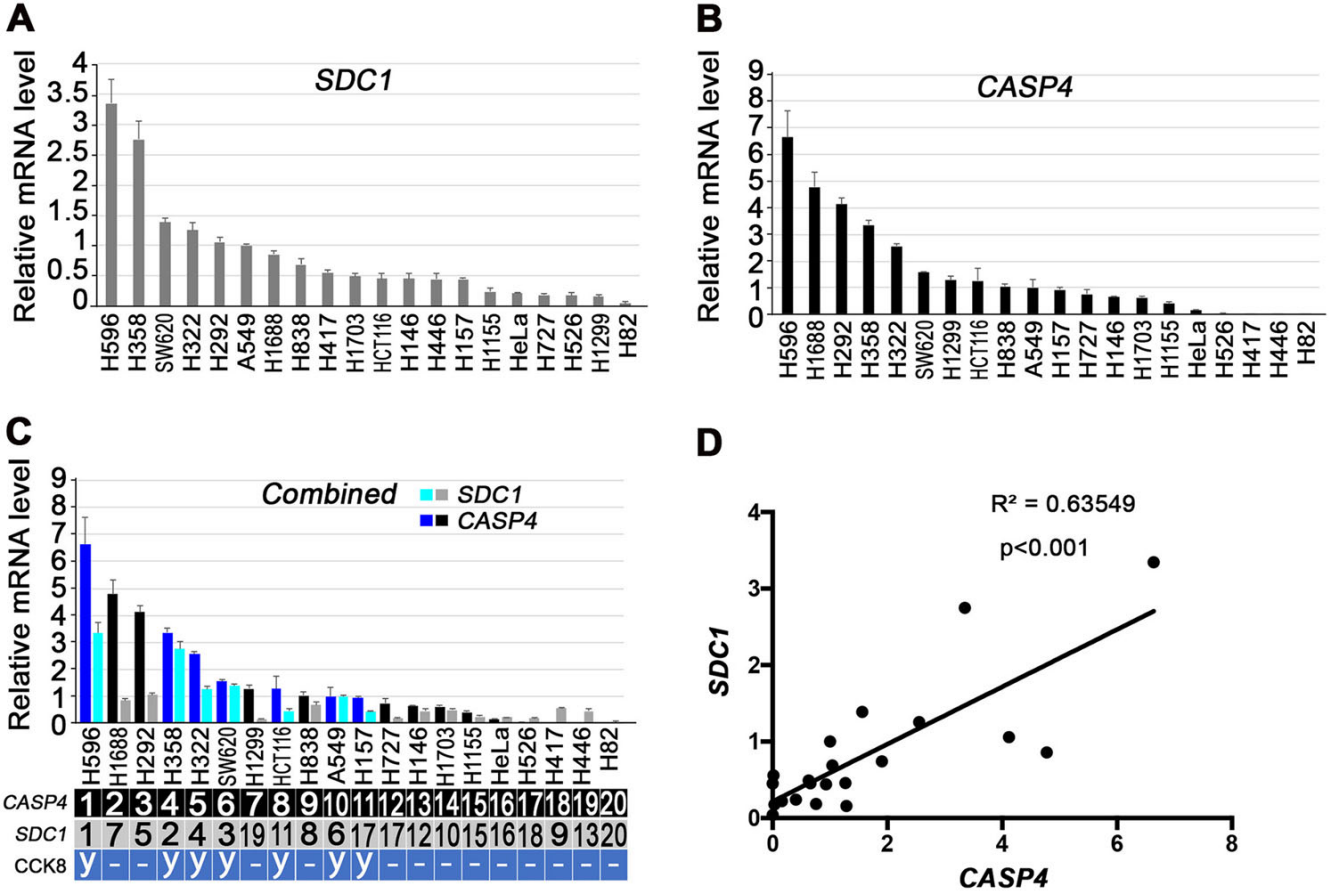
Evaluation of the susceptibility to SCGB3A2-LPS and expression analysis of SDC1 and CASP4 in various human cancer cells. **A**, **B** qPCR quantification of the relative expression levels for *SDC1* (**A**) and *CASP4* mRNA (**B**) in various human malignant cells. The expression level in A549 cells was arbitrarily set as 1.0. Graphs are representative of three independent experiments, each done in triplicate. **C** Combined qPCR results of **A** and **B**. Ranked order for *CASP4* and *SDC1* mRNA expression is indicated in the order from the highest to lowest, shown underneath the graph. The susceptibility to SCGB3A2 + LPS determined by CCK8 assay is shown as “y (observed)” or “– (not observed)“ in the CCK8 row. A bar for *CASP4* and *SDC1* mRNA of those susceptible to SCGB3A2 + LPS is indicated by dark blue (*CASP4*) and light blue (*SDC1*), respectively. **D** Correlation graph between *SDC1* and *CASP4* mRNA levels in 20 human cancer cells. Pearson correlation *r* = 0.7988, *P* < 0. 0001.

Previously, we showed that LLC cells have high expression of SDC1 and CASP11, with strong susceptibility to SCGB3A2-induced anti-tumor activity, whereas B16F10 melanoma cells have very little expression of SDC1 and CASP11, and no susceptibility to SCGB3A2^17^. This indicates that the SDC1/CASP11 expression patterns could determine the susceptibility of cancer cells to SCGB3A2-induced anti-tumor activity. To examine if this correlation is also found in human cancer cells, a panel of 20 human cancer-derived cell lines (17 lung, 2 colon, and 1 cervix) were analyzed for the constitutive expression of mRNA encoding SDC1 and CASP4 (CASP4 and CASP5 are the human equivalent of CASP11 in mice) (Fig. 1A–C). No significant expression of *CASP5* mRNA was detected in all human cancer cell lines examined (data not shown), and therefore we focused on CASP4 in this study. A highly positive correlation was obtained between the expression levels of *SDC1* and *CASP4* mRNAs, suggesting an interaction at the genomic and/or genetic levels between the SDC1 and CASP4 (Fig. 1C, D).

### Cell surface expression of SDC1 and its component HS, and CASP4 are critical for SCGB3A2-induced growth inhibition of cancer cells

**Fig. 2:**
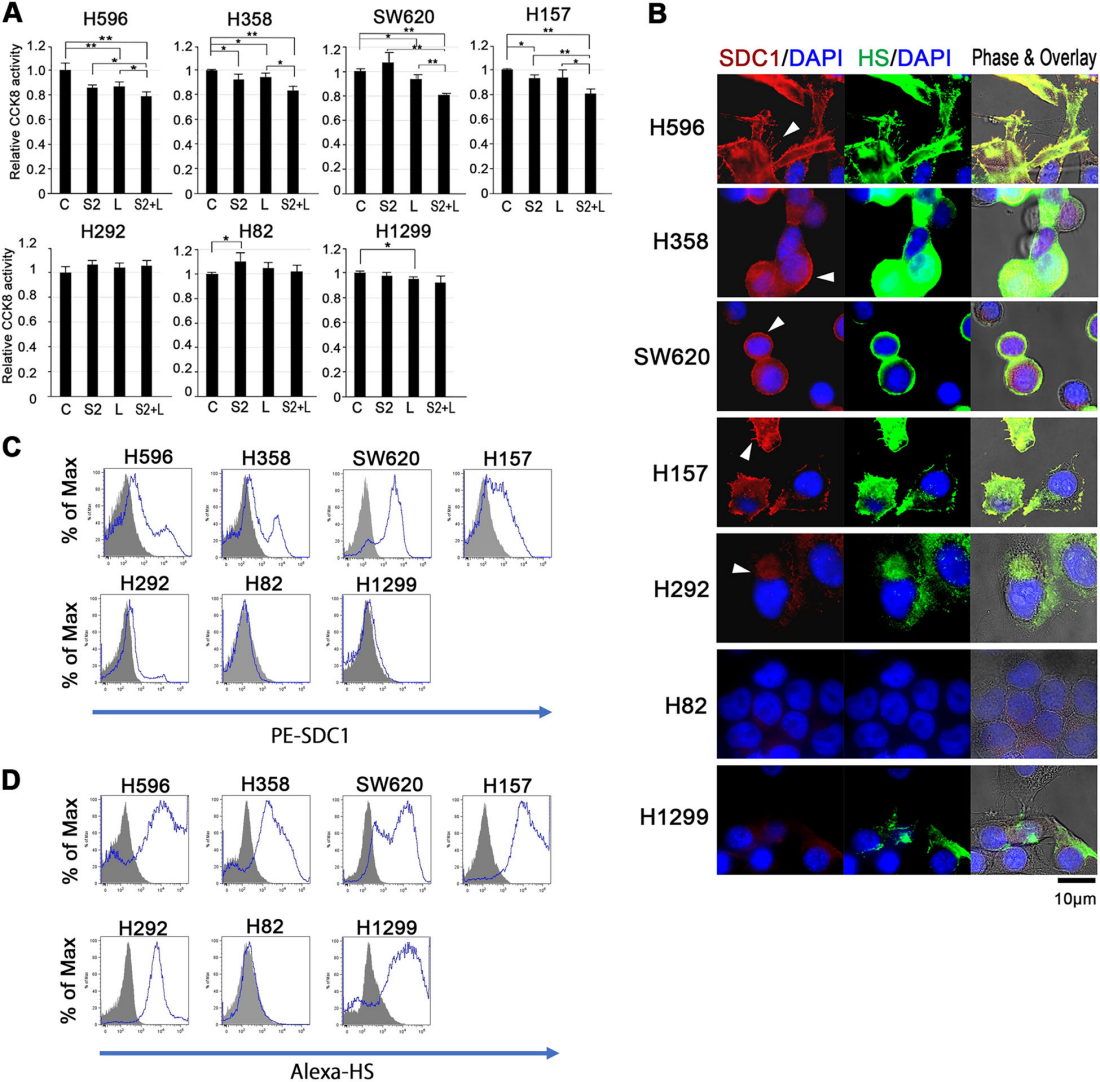
Examination of human cancer cells for cell growth and expression pattern of SDC1 and HS. **A** Representative CCK8 analysis results. Average ± SD from more than three independent experiments. C, control; S2, SCGB3A2; L, LPS. *P < 0.05, **P < 0.01 by one-way ANOVA. **B** Representative immunofluorescence analysis. Counter stained with DAPI. Bar = 10 µm. White arrowheads indicate the membranous SDC1 expressions. **C**, **D** Flow cytometric analysis for SDC1 and HS expression on cell surfaces of human malignant cells using anti-SDC1 (**C**) and anti-HS antibody (**D**). Gray histograms indicate unstained negative control. Experiments were carried out more than three times and at each time, similar results were obtained.

To evaluate the effect of SCGB3A2 with and without additional LPS on their cell growth in vitro, CCK8 assays were carried out (Fig. 2A). Of the 13 human cancer cell lines examined, 5 NSCLCs (NCI-H596, H358, H322, A549, and H157) and 2 colorectal cancers (HCT116 and SW620) showed approximately 20% growth reduction by SCGB3A2 + LPS (Figs. 1C and 2A and Supplementary Table S1). They are all epithelial-derived cell lines. In contrast, the small cell lung cancer cells (SCLCs) analyzed (NCI-H526, H417, H146, H446, H82, and H1688), which were derived from a rare population of pulmonary neuroendocrine cells^18^, exhibited no obvious responses to SCGB3A2 + LPS (Figs. 1C and 2A for H82 and Supplementary Table S1, data not shown). To further understand the underlying mechanism for the differences in response to SCGB3A2 + LPS, the expression patterns of SDC1 receptor and HS, the main glycosaminoglycan chain attached to the extracellular core protein of the syndecan family of proteins, were evaluated by immunofluorescence (Fig. 2B, Supplementary Fig. S1 and Supplementary Table S1) and flow cytometry analysis for their cell surface expression levels (Fig. 2C, D, Supplementary Fig. S2 and Supplementary Table S1). Immunofluorescence analysis revealed that all seven of the SCGB3A2-susceptible human cancer cells expressed SDC1 on their cell surfaces (Fig. 2B for H596, H358, SW620, and H157 and Supplementary Fig. S1D, F, K for H322, A549, and HCT116, white arrowheads). In contrast, non-susceptible cells showed little SDC1 expression (Fig. 2B for H82 and Supplementary Fig. S1I, L, R for H417, H146, and H526) or showed cytoplasmic/nuclear/perinuclear SDC1 expression (Fig. 2B for H292, white arrowhead, Supplementary Fig. S1G, J, M and S1Q for H1688, H1703, H446, and H727). Interestingly, H1299 cells with very little SDC1 but abundant HS expression (Supplementary Fig. S1S) were not susceptible to SCGB3A2 + LPS treatment (Figs. 1C and 2A). Further, H1155 cells did not respond to SCGB3A2 + LPS (Fig. 1C and Supplementary Table S1) that have membranous SDC1 expression by both immunofluorescence and flow cytometric analysis without detectable levels of HS (Supplementary Figs. S1O and S2O). These results suggest that SCGB3A2 appears to interact with the HS moiety of SDC1. Our previous results demonstrated that SDC1 expression and SCGB3A2 + LPS when added to the culture medium of LLC cells, localize to uropods^17^, known to be concentrated at cell-to-cell contact points or junctions and accumulate growth factors^19,20^, and SCGB3A2 + LPS interacts with SDC1 though the HS moiety^17^. These results indicate that the synergistic high-level expression of both SDC1 with specific HS sequence at the cell membrane and CASP4 are critical for SCGB3A2 + LPS effects on the growth inhibition of cancer cells.

### SCGB3A2-induced growth inhibition of cancer cells is due to pyroptosis

**Fig. 3:**
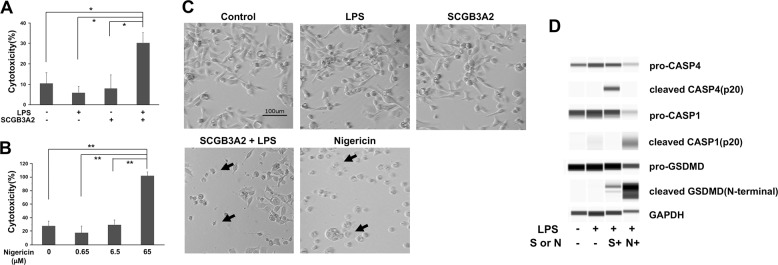
Analysis of non-canonical inflammasome pathway. **A** LDH cytotoxicity assay in the presence of LPS or SCGB3A2 alone, or the two together (SCGB3A2 + LPS). **B** LDH cytotoxicity assay in the presence of various amount of nigericin. Average ± SD from more than three independent experiments. *P < 0.01, **P < 0.0001 by Tukey’s multiple comparison. **C** Morphology of cells cultured in the presence of LPS or SCGB3A2 alone, SCGB3A2 + LPS, or nigericin for 3 h. For nigericin-treated group, cells were primed with LPS before addition of nigericin. Black arrow indicates ballooned cells, characteristic feature of pyroptosis. **D** Western blotting for the cleaved forms of CASP1 (p20), CASP4 (p20), and GSDMD (N-terminal). S: SCGB3A2, N: nigericin. Experiments were repeated more than twice and same results were obtained.

In order to demonstrate that SCGB3A2 together with LPS activates pyroptosis that results in inhibited growth of human cancer cells as previously shown for mouse LLC cells^17^, H596 cells were subjected to lactose dehydrogenase (LDH) cytotoxicity assay (Fig. 3). H596 cells were chosen because these cells exhibited the highest mRNA levels for both *SDC1* and *CASP4* (see Fig. 1). LPS and SCGB3A2 exhibited increased LDH activity in the media of H596 cells, as compared with control, or addition of LPS or SCGB3A2 only (Fig. 3A). The degree of cytotoxicity was SCGB3A2 + LPS incubation time dependent (Supplementary Fig. S3). LDH is a cytosolic enzyme involved in energy production, and the detection of the activity in media means that LDH leaked out cells because of cell damages, indicative of pyroptosis. Nigericin known to induce caspase-1-dependent pyroptosis^21^ was used as a control (Fig. 3B). In fact, cells having characteristic appearance of pyroptosis (ballooning) were found in SCGB3A2 + LPS as well as nigericin-treated cells (Fig. 3C). Western blotting showed that cleaved CASP4 as well as gasdermin D (GSDMD) bands were seen only in cells treated with SCGB3A2 + LPS where no CASP1 activation was found, whereas nigericin-treated cells produced cleaved CASP1 and GSDMD bands (Fig. 3D and Supplementary Fig. S4). These results demonstrate that the SCGB3A2 + LPS-induced pyroptotic death of H596 cells is the result of activated non-canonical inflammasome pathway through CASP4.

### SCGB3A2 limits the growth of human NSCLC and colorectal cancer cells in the mouse metastasis model

**Fig. 4:**
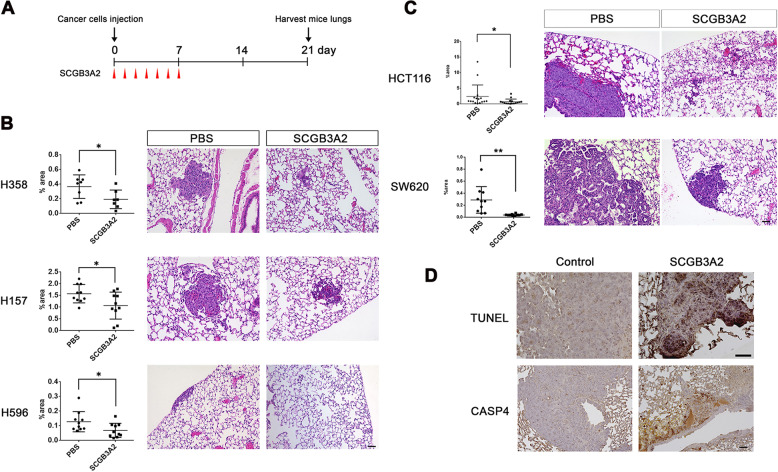
Inhibition of human cancer cells growth in vivo by SCGB3A2. **A** Human cancer cells intravenous metastasis mouse model scheme. **B**, **C** Summary for the total tumor area of lung inoculated with human NSCLCs; NCI-H358, H157, H596 (**B**), and human colorectal cancer cells; HCT116 and SW620 (**C**). Averages ± SD from *N* = 5 per group for lung cancer cells and SW620, *N* = 8 for HCT116. Two sections at different positions of tissue per mouse were used for the analysis. *P < 0.05, **P < 0.01. Representative HE staining images of tissue sections from each PBS or SCGB3A2 administration group are shown on the right. Bar = 50 μm. **D** Representative images of TUNEL and CASP4 staining of lung sections of lung metastasized HCT116 cells from control (PBS) and SCGB3A2 administered mice. Three independent tissue samples were evaluated for each group, and similar results were obtained. Bar = 50 μm.

To evaluate the effects of recombinant SCGB3A2 on the development of human NSCLC cells in vivo, three NSCLC cell lines (H596, H358, H157) susceptible to SCGB3A2 + LPS by CCK8 assay in vitro (see Fig. 2A) were subjected to mouse intravenous xenograft experiments (Fig. 4A). SCGB3A2 significantly reduced the growth of all three NSCLC cells (Fig. 4B) in the mouse metastasis model as determined by a percentage of tumor area per total area. These results indicate that SCGB3A2 may reduce the growth of NSCLC tumor cells.

Two human epithelial-derived colorectal cancer cells (HCT116 and SW620) also showed susceptibility to SCGB3A2 + LPS by CCK8 assay in vitro (Figs. 1C and 2A and Supplementary Table S1). In the metastasis mouse model, both HCT116 and SW620 cells showed similar susceptibility to SCGB3A2 (Fig. 4C). They both express high levels of *CASP4* and SDC1/HS on their cell surface (Fig. 2B–D and Supplementary Figs. S1K and S2K). Furthermore, TUNEL analysis demonstrated that SCGB3A2 caused high levels of cell death of lung-metastasized colorectal cancers (Fig. 4D for HCT116, data not shown). Immunohistochemistry showed that these colorectal tumor nodules expressed CASP4 in lung metastases, which was enhanced by SCGB3A2 administration and correlated with the inhibition of tumors (Fig. 4D for HCT116, data not shown). These results indicate that SCGB3A2 may also inhibit growth/metastasis of intestine epithelial originated cancer cells, by means of the CASP4-mediated pyroptosis pathway.

### SCGB3A2 expression may be a good prognostic marker of lung adenocarcinomas

**Fig. 5:**
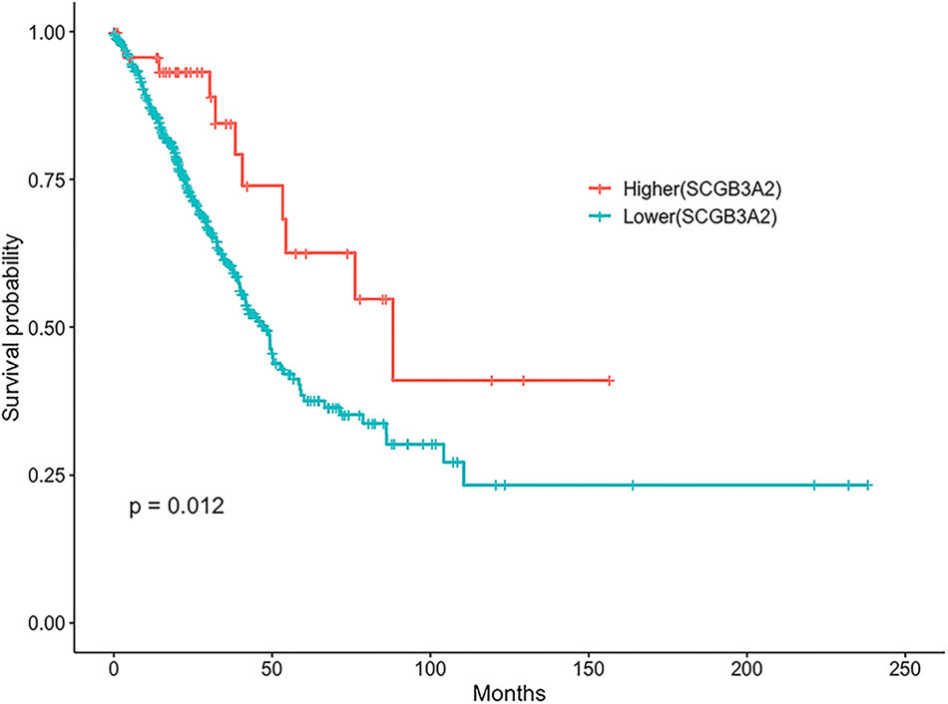
TCGA data analysis. Survival curve for lung adenocarcinoma patients (*n* = 494) expressing higher *SCGB3A2* (red, *n* = 49, top 10%) or lower *SCGB3A2* (blue, *n* = 445, remaining 90%).

The Cancer Genome Atlas (TCGA) database was used to analyze the expression of SCGB3A2 among various lung cancers. The survival rate of lung adenocarcinoma patients expressing high levels of SCGB3A2 was higher than those with low expression (Fig. 5). Further, among all lung adenocarcinoma patients analyzed, three patients expressed high levels (upper 20%) of all three critical genes *SCGB3A2*, *SDC1*, and *CASP4*, and all of them survived up to 40 months, although the “*n*” number is too small to establish significance (Supplementary Fig. S5A). The results suggest that SCGB3A2 may be a good prognostic marker for lung adenocarcinomas.

## Discussion

Pyroptosis has been mainly studied using innate immune cells such as mouse bone marrow-derived macrophages as a model. An increasing number of studies describe the role of pyroptosis in other cell types including epithelial cells and cancer cells^22–24^. In recent years, the pyroptosis of cancer cells as a cancer treatment has received some attention^25,26^; however, it remains largely unexplored. We found that SCGB3A2-delivered cytosolic LPS activates non-canonical inflammasome pathway in human cancer cells when they have high expression of membranous SDC1/HS and CASP4, leading to pyroptotic death; five NSCLC and two colon cancer cells were susceptible to SCGB3A2 + LPS treatment in vitro and in vivo, resulting in growth suppression of cancer cells.

It is notable that both NSCLC and epithelial-derived colorectal cancer cells showed strong responses to SCGB3A2. Colorectal epithelial cells, like lung airway epithelial cells, represent another frontline barrier at sites of high exposure to various pathogens entering the body. While lung airway incorporates various pathogens from the air, intestinal epithelial cells are constantly exposed to a multitude of microorganisms entering the host, food derived pathogens, and endotoxins^27^. It seems that the machinery to fight against microorganisms and infection triggering non-canonical inflammasome pathway has been retained in some cancer cells. Indeed, in our previous work, we showed that macrophage-derived RAW264.7 cells express SDC1 and are susceptible to SCGB3A2-induced pyroptosis^17^. The results highlight the importance of SCGB3A2 in some cancer cells to trigger their pyroptotic cell death, demonstrating a promising function of SCGB3A2 as an inhibitor of cancer cell growth. Further notable is that none of SCLCs analyzed, exhibited high levels of membrane SDC1 expression, and they were refractory to SCGB3A2 + LPS. Our tumor xenograft model using intravenous injection of SCLC cells failed to develop visible tumor burdens (data not shown). We may need to extend the observation period much longer than 21 days with this model or use a different xenograft model such as subcutaneous injection in order to determine the effect of SCGB3A2 on SCLC tumor development.

Endotoxin (LPS) is normally considered to be a pro-inflammatory molecule. It is also well recognized that persistent, or exaggerated inflammation is related to cancer growth and metastasis. However, paradoxically, some experiments suggest the counterintuitive effects of endotoxin to inhibit cancer growth^28^. Since the 1970s, epidemiological studies of endotoxin-exposed occupational groups such as cotton textile have consistently demonstrated significantly reduced lung cancer risks of these workers^28^. Treatment of human cancer by directly injecting bacterial endotoxin dates back to the treatment by William. B. Coley^29–31^. It was believed that his treatment using killed bacteria (Coley’s toxin) could boost activities of innate immune cells, resulting in the spontaneous regression of tumors. This is regarded as one of the first examples of an immunotherapy. However, as our results demonstrate, some cancer cells undergo pyroptosis in vivo upon exposure to cytosolic LPS delivered by SCGB3A2, in addition to its immunomodulatory function. Our previous study and the present work suggest that a very small amount of LPS, present in the body (~0.05 ng/ml in serum^32^), is sufficient to interact with SCGB3A2 when administered, and activate pyroptosis of some cancer cells both in humans and mice.

TCGA analysis results support the notion that SCGB3A2 suppresses the growth of cancer cells in humans, particularly lung adenocarcinomas. It was reported that *SCGB3A2* has a tendency to be downregulated in lung squamous cell carcinoma with metastasis compared with non-metastatic disease; however, no statistical differences found^33^. Further, TCGA data suggest that some colon cancers may acquire the expression of SCGB3A2 because colon is not an organ where *SCGB3A2* expression is usually found in normal tissue (our own observation and the Human Protein Atlas: https://www.proteinatlas.org/ENSG00000164265-SCGB3A2/tissue). Whether this is indeed the case awaits further studies. It is interesting to note that among the top ~50% of *SDC1* and among which the top 20% *CASP4* high-expressing lung adenocarcinoma and colon cancer patients (out of 503 for lung and 524 for colon, respectively), approximately 20 (lung) and 50 (colon)% of them possess *KRAS* mutations (Supplementary Tables S2 and S3). Currently no approved targeted therapy is available for *KRAS* mutated cancers^34^. SCGB3A2-LPS treatment may provide a tool to treat this group of cancer patients.

TCGA human lung and colon cancer databases showed that the mutation rate of *CASP4*/*5* is rare (6–9%) as previously described^35^ (Supplementary Fig. S5B, C). This is in sharp contrast to p53 (*TP53*) gene, the key regulator of programmed cell death that is known to be most frequently mutated in human cancers (overall, about 50% of all cancers have p53 mutant alleles) and one of the best studied genes involved in human cancers^36–38^. Several studies demonstrated that restoration of wild-type p53 function can suppress tumor growth, so reactivation of mutant p53 to wild-type form was attempted as a strategy to treat cancer^37–39^. However, it has not been successful largely because mutations in p53 are predominantly missense, or localized in the DNA binding domain, which are diverse, resulting in production of a large number of p53 mutants that have a unique set of quantitative defects in DNA binding, and/or transcriptional defects^38^. It is also known that some full-length forms of mutant p53 found in tumors have acquired oncogenic activities, including increased proliferation, enhanced metastatic potential, and acquisition of resistance to targeted drugs^36–38^. Thus, the rare mutation rate and the ability to activate pyroptotic process of *CASP4* in the SCGB3A2-inducded non-canonical inflammasome pathway could be utilized as a new strategy for human cancer therapy.

In conclusion, SCGB3A2 uses the machinery of the pyroptotic cell death for the elimination of SDC1/CASP4-positive human cancer cells. This may be exploited as the potential use of this pathway in cancer treatment. SCGB3A2 may serve as a novel therapeutic to treat certain epithelial tumors, perhaps in combination with immuno and/or targeted therapies.

## Materials and methods

### Antibodies and reagents

Recombinant human (rh) SCGB3A2 and anti-human SCGB3A2 antibody used in this study were from APCBio Innovations (Rockville, MD)^40^. Anti-human SDC1 ectodomain antibodies were kindly provided by Dr. Pyong W. Park (Harvard Medical School). Anti-HS antibody (clone F58-10E4, 1:50 dilution) and anti-CASP4 antibody (PA5-94598, 1:100) for immunofluorescence/immunohistochemistry were purchased from Amsbio and Thermo Fisher Scientific, respectively. Antibodies used for western blotting were as follows: anti-CASP4 (#4450, recognizing p20 fragment, Cell Signaling Technology, 1:10), anti-CASP1 (#3866, recognizing p20 fragment, Cell Signaling Technology, 1:10), anti-gasdermin D (GSDMD) (ab155233, recognizing GSDMD N-terminal, Abcam, 1:50), and anti-GAPDH (NB100-56875, Novus Biologicals, 1:1000). LPS from *Escherichia coli* O111:B4 (E4391) and nigericin (SML1779) were obtained from Sigma-Aldrich and Cell counting kit-8 (CCK-8) reagent from Dojindo.

### Cell culture

Human lung cancer-derived cell lines, A549, NCI-H322, H358, H1299, H146, H596, H82, H526, H417, H446, H727, H292, H1155, H157, and H1688 cells were obtained from Curt Harris (NCI), H838 and H1703 cells were from Giuseppe Giaccone (then NCI, currently Georgetown University), and SW620 colon cancer derived cell line was from the DTP (Developmental Therapeutics Program, NCI/DCTD (Division of Cancer Treatments and Diagnostics)) tumor cell lines depository. HeLa and HCT116 cells were purchased from American Type Culture Collection (ATCC).

Most of human cell lines were cultured in RPMI 1640 Medium (LONZA) with or without heat-inactivated fetal bovine serum (FBS: GEMINI BIO), supplemented with penicillin/streptomycin (1:100) or antibiotic/antimycotic solution (10,000 units/ml penicillin G, 10 mg/ml streptomycin sulfate, 25 μg/ml amphotericin B: GEMINI BIO) at 37 ˚C, 5% CO_2_. HCT116 cells were cultured in McCoy’s 5A Medium (LONZA) instead of RPMI1640 as a medium. For in vitro CCK8 assays, rhSCGB3A2 (1 μg/ml) containing 0.00936 EU (endotoxin unit)/μg of LPS^17^ was mixed with 100 pg/ml of LPS (10:1 weight ratio for rhSCGB3A2/LPS) and incubated for 10 min at room temperature prior to addition to the culture media.

### Quantitative RT-PCR

Total RNA was extracted by TRIzol® (Life Technologies) and reverse transcribed into cDNA by using SuperScript III reverse transcriptase (Life Technologies) according to the manufacturer’s protocol. Analysis of mRNA levels was performed on a 7900 Fast Real-Time PCR System (Life Technologies) with SYBR Green-based real-time PCR. The primer sequences used for real-time PCR are as follows:

(sense) 5′-ACAAGACCCACGTGGAGAAG-3′ and

(antisense) 5′-AGCCCATTGTGCTGTCTCTC-3′ for human *CASP4*;

(sense) 5′-TCCTGGACAGGAAAGAGGTG-3′ and

(antisense) 5′-TACAGCATGAAACCCACCAG-3′ for human *SDC1*

(sense) 5′-CTGACTTCAACAGCGACACC-3′ and

(antisense) 5′-TGCTGTAGCCAAATTCGTTG-3′ for human *GAPDH*.

The relative mRNA levels were plotted based on that of A549 cells arbitrarily set as 1.0, whose *CASP4* expression level sit at the middle (10th) among all 20 cells examined.

### Fluorescence-activated cell sorting (FACS) analysis

For SDC1 and HS expression analysis, human cancer cells were harvested in phosphate-buffered saline (PBS) and stained with each specific antibody (1:100) for 30 min. After washing with PBS several times, cells were stained with PE-conjugated secondary antibodies (BD Biosciences, 1:200) for 30 min, followed by washing with PBS. Cells were analyzed in a FACS Canto II (Becton Dickinson). All experiments were carried out at 4 ˚C and analyzed in the NCI Flow Cytometry Core Facility according to the facility’s instruction.

### Immunofluorescence analysis

All adherent cells were seeded on glass coverslips (Nunc™ Lab-Tek™ Chambered Coverglass, 15583PK). Floating cells in culture were subjected to cytospin (Shandon) to immobilize cells on glass slides (Shandon™ Double cytoslides™) before further process. Cells were fixed with 10% buffered formalin for 10 min at room temperature (RT), followed by permeabilization with 100% MeOH at −20 ˚C for 10 min. Blocking was done with 1% BSA in PBS for 1 h and cells were stained with primary antibodies for 1 h at RT, washed with PBS, and were stained with secondary antibodies (1:200, Alexa flour, Molecular Probe) for 45 min at RT. Cell nucleus were counter stained with 4′,6-diamidino-2-phenylindole for 10 min at RT. Cells were analyzed under a Keyence microscope BZ-X700.

### LDH assay

Cells were seeded in a 96-well plate (5 × 10^4^ cells/well), cultured for 24 h, and then the medium was changed to that containing 2% FBS. Lactate dehydrogenase (LDH) measurement was carried out after cells were cultured in the presence of LPS (25 μg/ml) and/or rhSCGB3A2 (25 μg/ml), or Nigericin (concentration as indicated) for 6 h. For Nigericin treatment, cells were pre-cultured with LPS (1 μg/ml) for 4 h as priming. Cell supernatants were evaluated for the presence of cytoplasmic enzyme LDH using the Cytotoxicity LDH Assay Kit-WST (Dojindo, Kumamoto, Japan). Cytotoxicity was calculated according to the manufacturer’s instruction: as a percentage of (experimental LDH−spontaneous LDH)/(maximum LDH release−spontaneous LDH).

### Western blotting

Cells seeded in six-well plate (2 × 10^6^ cells/well) were cultured for 24 h and then the media were changed to those of FBS-free. LPS and rhSCGB3A2 (1:1 by weight) were incubated for 30 min at room temperature before adding to the cells. Cells were treated with LPS (50 μg/ml) and/or rhSCGB3A2 (50 μg/ml), or Nigericin (65 μg/ml) for 4 h and cells and culture supernatants were separately collected. The cell lysate was prepared using RIPA buffer. In the Nigericin-treated group, cells were cultured with LPS (10 μg/ml) for 3 h as priming before Nigericin treatment. For cleavage studies (Caspase-4 p20, Caspase-1 p20, and cleaved GSDMD N-terminal), culture supernatants were precipitated by 10% trichloroacetic acid and 0.05% sodium deoxycholate and reconstructed in RIPA buffer. Immunoblotting was performed by the WES automatic western blot system (ProteinSimple, San Jose, CA) using 12–230 kDa separation modules. Two micrograms of total protein from cell lysate or 400 μl of supernatant were loaded onto each capillary. Data were analyzed by Compass software (ProteinSimple).

### Human cancer cells mouse metastasis model

Human cancer cells (2 × 10^5^ cells) were intravenously administered via tail vein injection to NSG mice (Charles River, Frederick, MD, 6–8 weeks old, males and females combined), followed by daily intravenous administration of rhSCGB3A2 (0.25 mg/kg/day) for 7 days starting at day 0 (30 min after cancer cells injection) or PBS injection as control. Mice were killed on day 21. All lungs were subjected to histological analysis and the total size of metastatic lung tumors (% of total area) was quantified using Hybrid Cell Count/BZ-H3C software (Keyence). For each specimen, two different sections of the slides (1/4 and 1/2 position from the peripheral surfaces) were evaluated for calculating a total percentage of tumor regions. Eight mice were used for HCT116, and five mice were used for the other cell lines. No randomization nor blinging was used in this study. All animal studies were carried out after approval by the National Cancer Institute Animal Care and Use Committee.

### Histological analysis

Lung samples were fixed in 10% buffered formalin under 20-cm H_2_O pressure, embedded in paraffin. Sectioning (4 μm by microtome) and hematoxylin and eosin (H&E) staining were carried out by Histoserv (Germantown, MD).

### TUNEL assay

Terminal deoxynucleotidyl transferase-mediated dUTP-biotin nick end labeling (TUNEL) analysis was performed using DeadEnd™ Fluorometric TUNEL System (G3250; Promega) according to the manufacturer’s instructions. The tissue was counterstained with hematoxylin.

### Immunohistochemistry

Lung sections were deparaffinized, rehydrated with graded EtOH series, and washed with H_2_O. Inactivation of endogenous peroxidase was performed with 3% hydrogen peroxide for 10 min. After blocking with 1% BSA, sections were stained overnight with anti-CASP4 antibody, washed with PBS, and then stained with HRP-conjugated secondary antibody for 1 h. After washing with PBS, immunostaining was carried out using DAB substrate kit (DAKO) with counterstaining with hematoxylin.

### TCGA data and survival analysis

The lung adenocarcinoma and colorectal adenocarcinoma data set (The Cancer Genome Atlas (TCGA)), which included 503 lung adenocarcinoma and 524 colorectal adenocarcinoma sequenced mRNA, mutation, copy number, and clinical data, were respectively downloaded from cBioPortal (http://www.cbioportal.org/)^41,42^. The top 10% of SCGB3A2-expressing patients were defined as “Higher (SCGB3A2)” group, while the rest were defined as “Lower (SCGB3A2)” group (see Fig. 5). “Higher (SCGB3A2)-higher (SDC1-CASP4)” group were defined as patients with expression of all three genes in top 20%, while the “Higher (SCGB3A2)-lower (SDC1-CASP4)” was defined as those among the top 20% SCGB3A2 expressors with the expression of neither SDC1 nor CASP4 being among the top 20%. (see Supporting Fig. S5A). Since Survival data were not available for all the patients, the number of patients used for this analysis was lower than those for the mutation and/or copy number analysis. Survival analysis was performed with the Kaplan–Meier method using the function “survfit” from package Survival (version survival_3.1–7, https://cran.rproject.org/web/packages/survival/index.html)^43^ of R (version 3.6.1, http://www.r-project.org/), and the figure was plotted by the function “ggsurvplot” from package Survminer (version 0.4.6, https://cran.r-project.org/web/packages/survminer/index.html) of R^44^. The OncoPrint for the *TP53*, *CASP4*, and *CASP5* were generated on cBioportal website with *z*-score 2^41,42^.

### Statistical analysis

Statistical analysis was carried out using GraphPad Prism v7. Data are shown as means ± SD. Levels of significance for comparison between samples were determined by student’s *t*-test or one-way ANOVA and Tukey’s multiple comparison. *P* values of < 0.05 were considered statistically significant.

## Supplementary information

Supplementary Figure Legends I would like to switch this figue legend to a new one since there are typos for gasdermin D (GSDMD).

Supplementary Table Legends

Figure S1

Figure S2

Figure S3

Figure S4

Figure S5

Table S1

Table S2

Table S3

## Data Availability

All data generated or analyzed during this study are included in this published article and its supplementary information files.
