# Supplementary material for: Secretoglobin 3A2 eliminates human cancer cells through pyroptosis
Source: Cell Death Discov. 2021 Jan 15;7:12. doi: 10.1038/s41420-020-00385-w (PMC7810848; doi:10.1038/s41420-020-00385-w)
Supplement: Supplementary file 1 — Supplementary Figure Legends I would like to switch this figue legend to a new one since there are typos for gasdermin D (GSDMD). [file 41420_2020_385_MOESM1_ESM.docx]

**Figure S1.** Representative immunofluorescence analysis for SDC1 and HS. Various human cancer cell lines were used as indicated. Counter stained with DAPI. White arrowheads indicate the membranous SDC1 expressions. Bar=10µm. *CASP4* mRNA levels are shown on the right as the value of A549 (F) arbitrarily set as 1.0.

**Figure S2.** Flow cytometric analysis for SDC1 and HS expression on cell surfaces. Various human cancer cells as indicated were analyzed using anti-SDC1 and HS antibody, respectively. Gray histograms indicate unstained negative control.

**Figure S3.** Time-dependent increase of LDH cytotoxicity under SCGB3A2+LPS in H596 cells. Average ± SD from more than 3 independent experiments. *P<0.05 by Tukey’s multiple comparison. Incubation time is hr.

**Figure S4.** Whole images of western blotting for CASP4, CASP1, GSDMD, and GAPDH. GAPDH was used as a loading control. S: SCGB3A2, N: nigericin. Arrows indicate pro-CASP4, CASP1, or pro-GSDMD. Arrowheads indicate their cleaved forms. Molecular marker bands with their molecular weights (kDa) are shown on the most left lane in each western results.

**Figure S5.** TCGA data analysis on the survival and mutation rates. (A) Survival curve for lung adenocarcinoma patients (n=494) expressing higher levels of *SCGB3A2, SDC1,* and *CASP4* genes (red, n=3, top 20% for *SDC1*, *CASP4* and SCGB3A2 high expression) or those of higher SCGB3A2 with lower *SDC1* and *CASP4* (blue, n=62). (B, C) The mutation rate of *CASP4* and *TP53* in lung adenocarcinoma (n=503) (B) and colorectal cancer patients (n=524) (C).
