# Supplementary material for: Secretoglobin 3A2 eliminates human cancer cells through pyroptosis
Source: Cell Death Discov. 2021 Jan 15;7:12. doi: 10.1038/s41420-020-00385-w (PMC7810848; doi:10.1038/s41420-020-00385-w)
Supplement: Supplementary file 2 — Supplementary Table Legends [file 41420_2020_385_MOESM2_ESM.docx]

**Table S1.** Summary of 20 human cancer cell lines for characteristics, gene expression patterns, and susceptibility to SCGB3A2. *1: qPCR, relative mRNA level compared with the value of A549 cell, *2: FACS mean, mean of subtraction of stained cells and unstained control. Representative graphs from 2-5 independent experiments were used. *3: FACS median, median of subtraction of stained cells and unstained control. Representative graphs from 2-5 independent experiments were used.

**Table S2.** List of gene mutations among lung adenocarcinoma patients with high *SDC1* and *CASP4* expression.

**Table S3.** List of gene mutations among colon cancer patients with high *SDC1* and *CASP4* expression
