# Supplementary figures and images for: Secretoglobin 3A2 eliminates human cancer cells through pyroptosis
Source: Cell Death Discov. 2021 Jan 15;7:12. doi: 10.1038/s41420-020-00385-w (PMC7810848; doi:10.1038/s41420-020-00385-w)

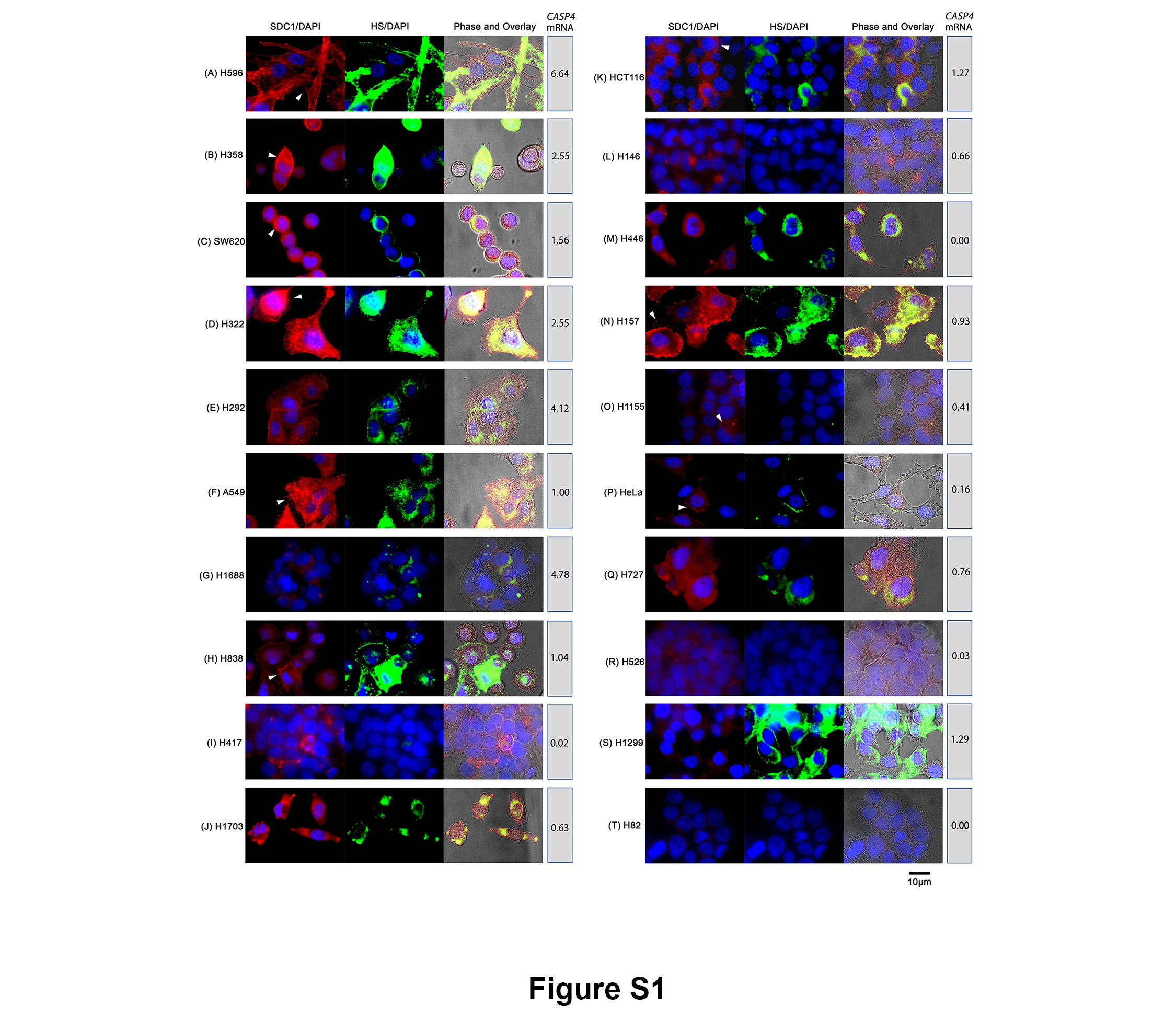

Supplement: Supplementary file 3 — Figure S1 [file 41420_2020_385_MOESM3_ESM.tif]

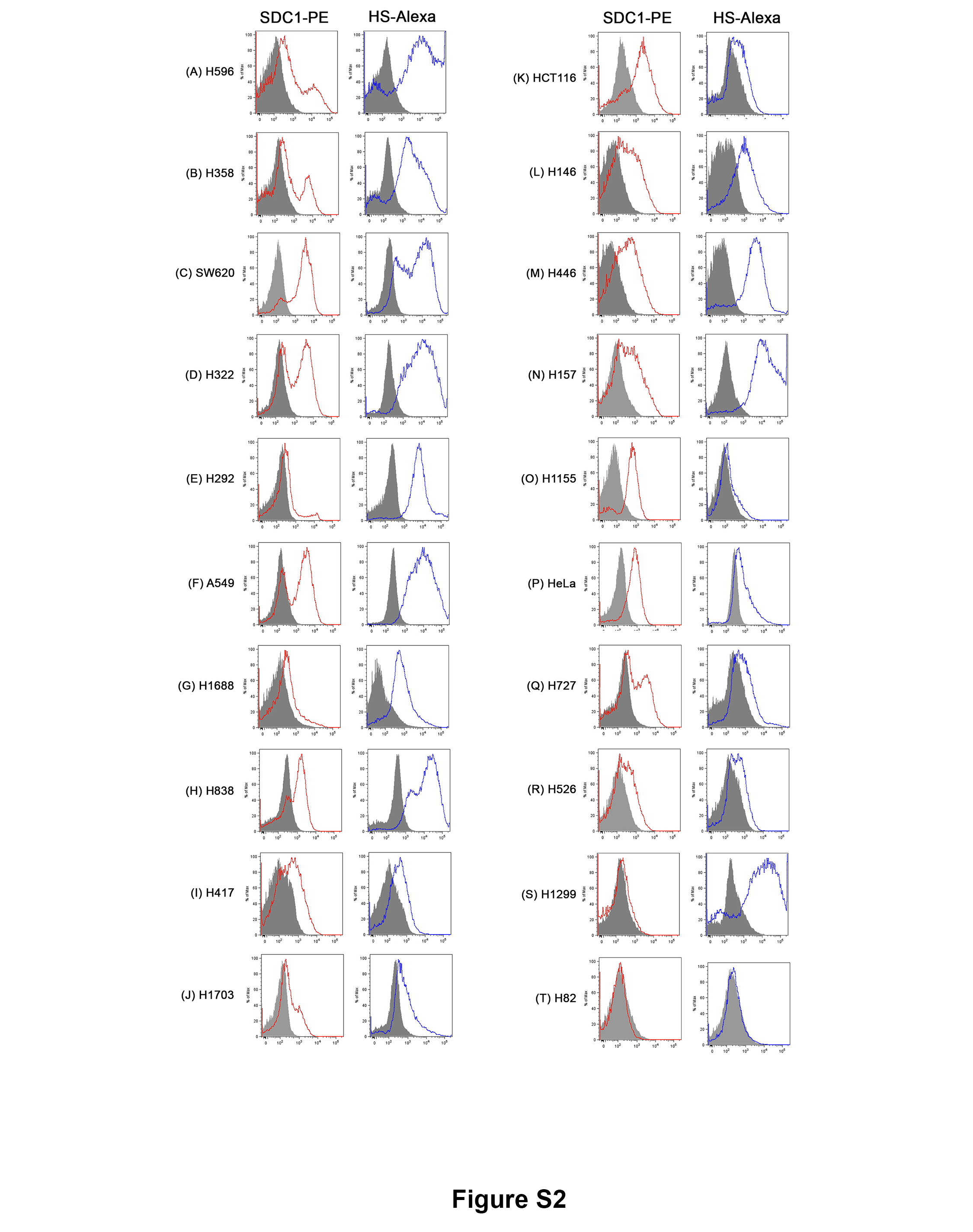

Supplement: Supplementary file 4 — Figure S2 [file 41420_2020_385_MOESM4_ESM.tif]

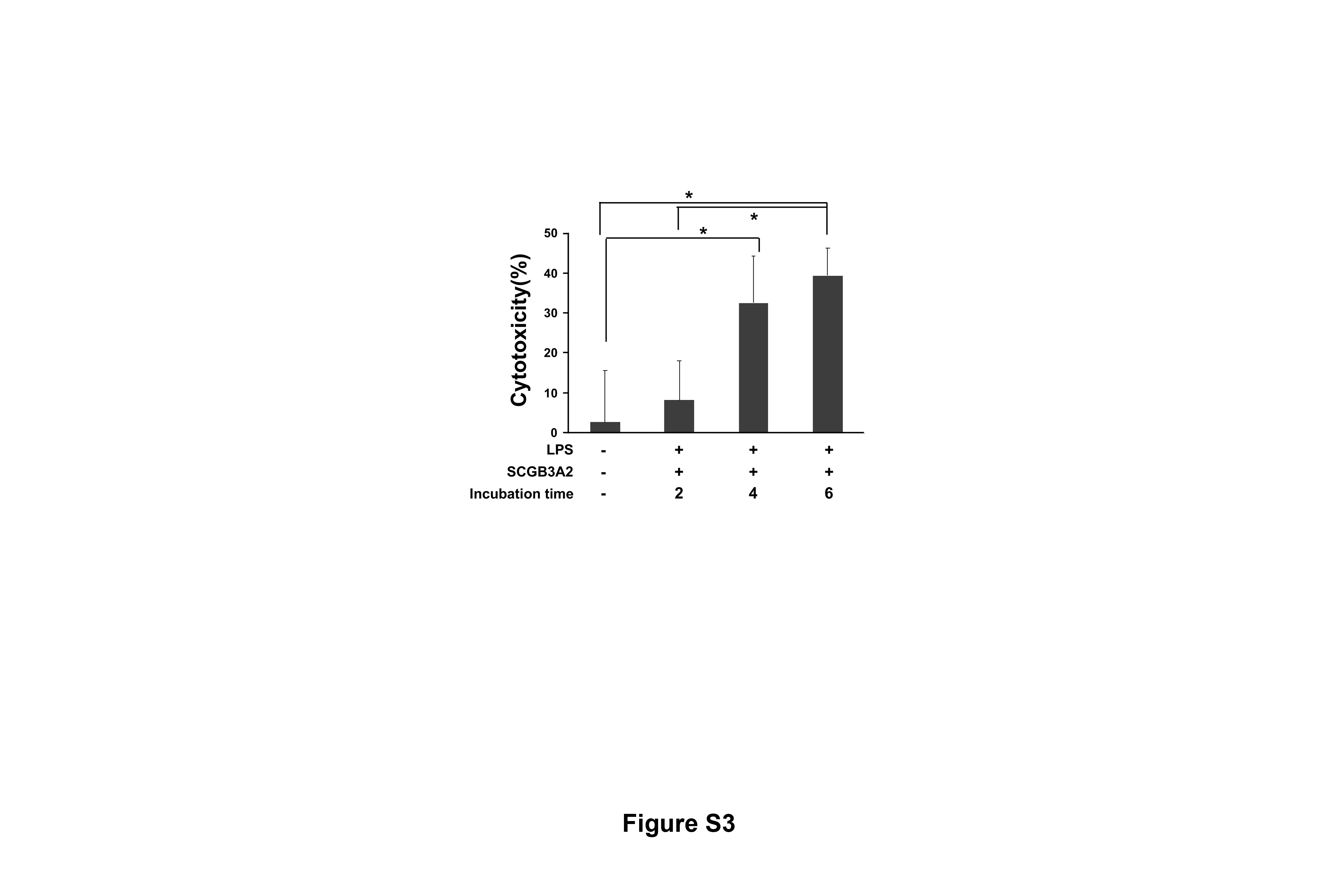

Supplement: Supplementary file 5 — Figure S3 [file 41420_2020_385_MOESM5_ESM.tif]

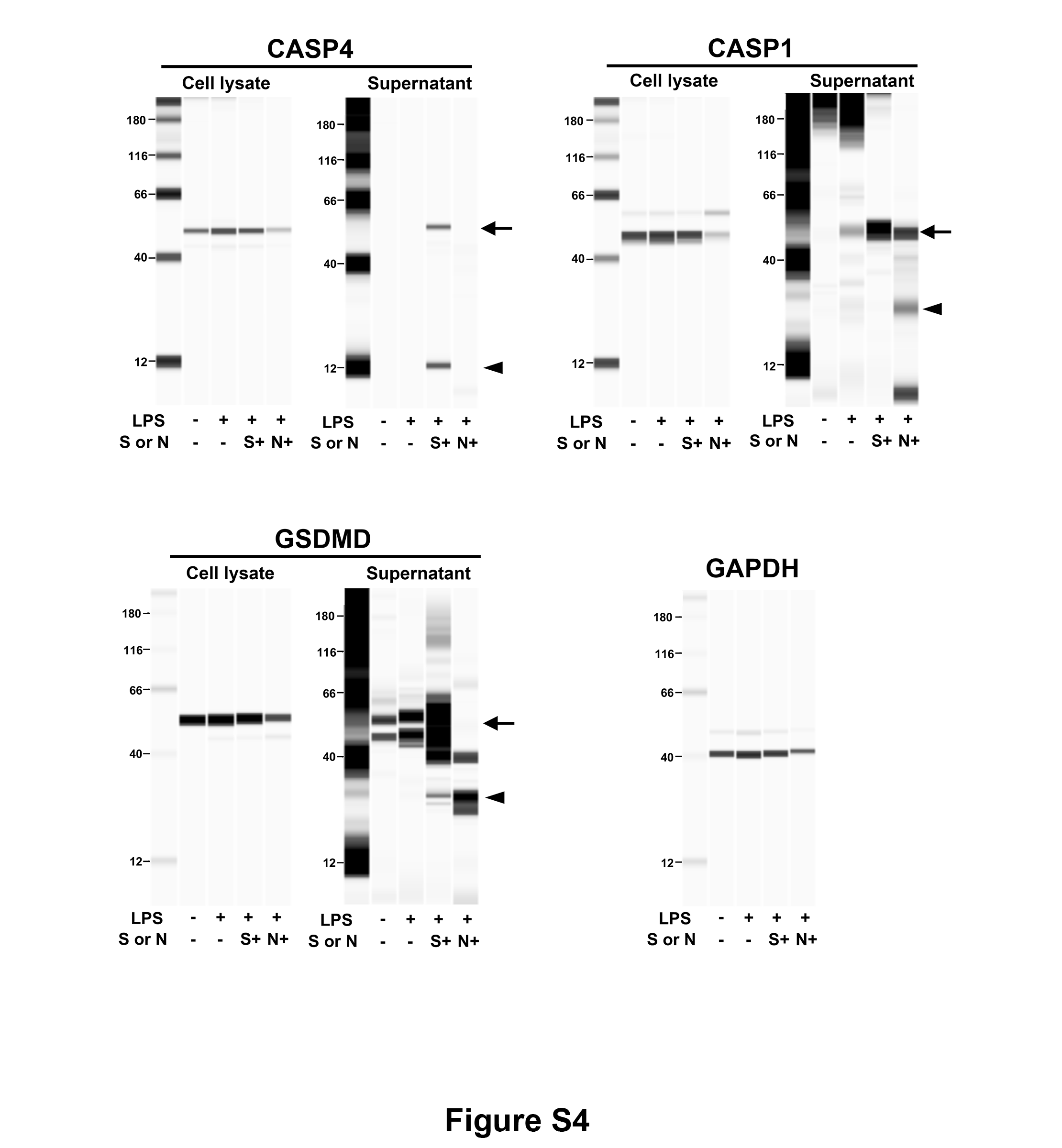

Supplement: Supplementary file 6 — Figure S4 [file 41420_2020_385_MOESM6_ESM.tif]

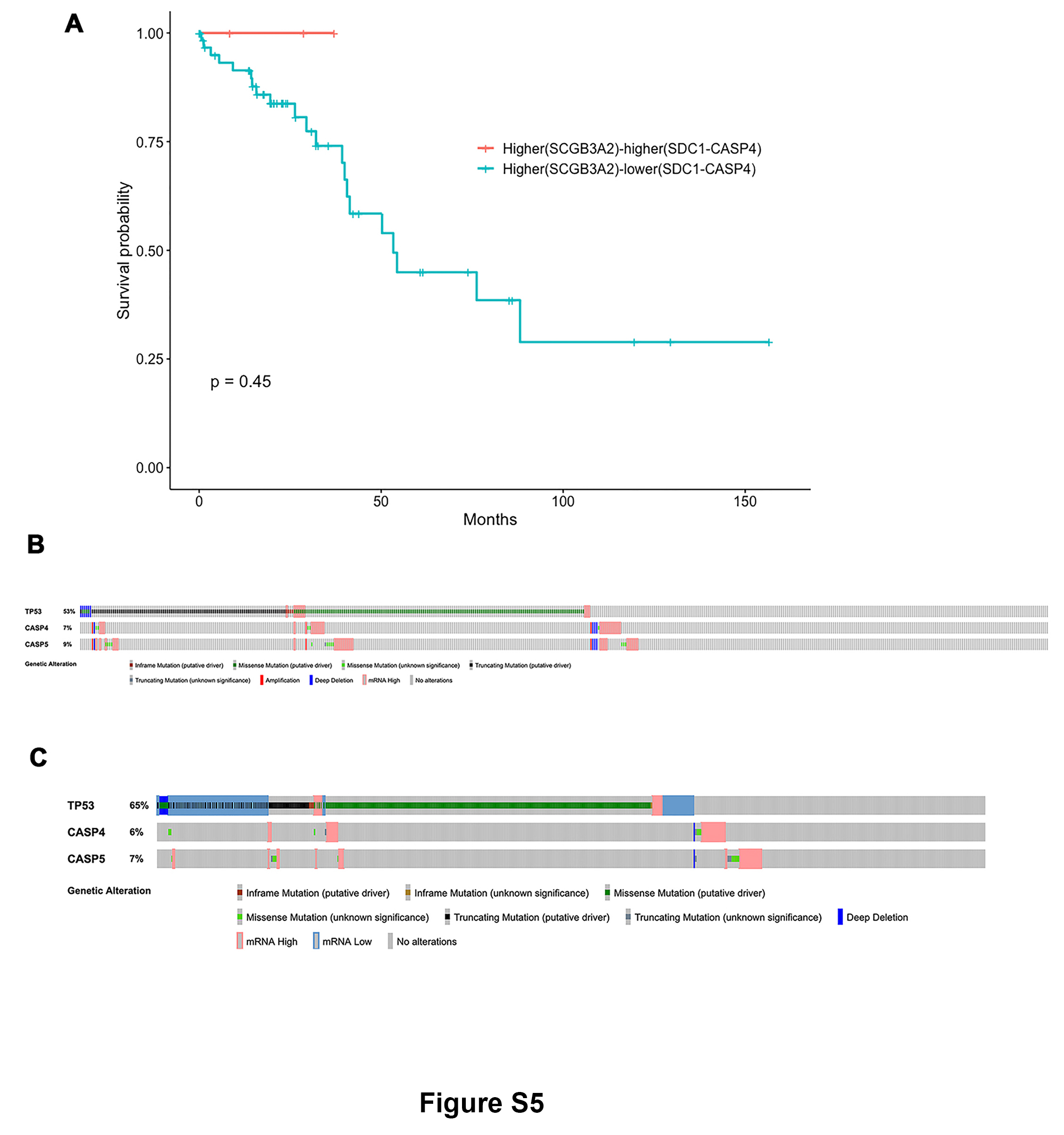

Supplement: Supplementary file 7 — Figure S5 [file 41420_2020_385_MOESM7_ESM.tif]
